# Volume and densities of chronic subdural haematoma obtained from CT imaging as predictors of postoperative recurrence: a prospective study of 107 operated patients

**DOI:** 10.1007/s00701-012-1565-0

**Published:** 2012-12-11

**Authors:** Milo Stanišić, John Hald, Inge Andre Rasmussen, Are Hugo Pripp, Jugoslav Ivanović, Frode Kolstad, Jarle Sundseth, Mark Züchner, Karl-Fredrik Lindegaard

**Affiliations:** 1 Department of Neurosurgery, Oslo University Hospital, Nydalen, Po. Box 4950, 0424 Oslo, Norway; 2 Department of Radiology, Oslo University Hospital, Oslo, Norway; 3 Intervention Centre, Oslo University Hospital, Oslo, Norway; 4 Unit of Biostatistics and Epidemiology, Oslo University Hospital, Oslo, Norway; 5 Faculty of Medicine, University of Oslo, Oslo, Norway

**Keywords:** Chronic subdural haematoma, Computerised tomography, Densities, Multiple regression, Recurrence, Volume

## Abstract

**Background:**

Chronic subdural haematoma (CSDH) is a common entity in neurosurgery with a considerable postoperative recurrence rate. Computerised tomography (CT) scanning remains the most important diagnostic test for this disorder. The aim of this study was to characterise the relationship between the recurrence of CSDH after treatment with burr-hole irrigation and closed-system drainage technique and CT scan features of these lesions to assess whether CT findings can be used to predict recurrence.

**Methods:**

We investigated preoperative and postoperative CT scan features and recurrence rate of 107 consecutive adult surgical cases of CSDH and assessed any relationship with univariate and multivariate regression analyses.

**Results:**

Seventeen patients (15.9 %) experienced recurrence of CSDH. The preoperative haematoma volume, the isodense, hyperdense, laminar and separated CT densities and the residual total haematoma cavity volume on the 1st postoperative day after removal of the drainage were identified as radiological predictors of recurrence. If the preoperative haematoma volume was under 115 ml and the residual total haematoma cavity volume postoperatively was under 80 ml, the probability of no recurrence was very high (94.4 % and 97.4 % respectively).

**Conclusions:**

These findings from CT imaging may help to identify patients at risk for postoperative recurrence.

## Introduction

Chronic subdural haematoma (CSDH), a disorder which mainly affects elderly individuals, is a common entity seen in routine neurosurgery. Surgical treatment usually results in rapid improvement of neurological symptoms and postoperative prognosis is generally good. However, the introduction of modern imaging methods and more sophisticated surgical strategies over the last few decades have not substantially improved a postoperative recurrence rate of 10–33 % [1] and therefore there is a need for more efficient treatment strategies to minimise relapse of CSDH. Identification of patients at high risk for relapse is particularly important because adjuvant treatment strategies should be directed toward them.

Computerised tomography (CT) scanning is the most important diagnostic test in CSDH. Numerous CT scan characteristics traditionally related to recurrence of CSDH have been reported and extensively discussed in the literature. These included bilateral site of CSDH [2–9], preoperative and postoperative haematoma width determined at the level of its maximum in-plane thickness [7, 8, 10–14], preoperative and postoperative maximum midline displacement [2, 7, 8, 10–12], haematoma densities [2, 4, 8, 13, 15–21], brain atrophy [19, 22–24], intracranial extension [17], enhancement and thickness of inner membrane of CSDH [25, 26], postoperative presence of residual air accumulation in the CSDH cavity [4, 7–9, 19, 27] and postoperative persistence of residual CSDH space [4, 9, 12, 27]. However, the findings are not consistent between the studies. Therefore we conducted a prospective investigation to characterise the relationship between the recurrence of CSDH after treatment with burr-hole irrigation and closed-system drainage and preoperative and postoperative CT scan features to assess whether CT findings can be used as prognostic factors for postoperative recurrence.

## Methods

The study was approved by the Regional Ethical Committee of Health Region South-East Norway (S-06281a) for the study of human subjects. Written informed consent was obtained from the patients or their close relatives before study inclusion.

### Patient population and management

We prospectively investigated preoperative and postoperative CT findings, and the postoperative recurrence in 107 consecutive adult patients operated on for CSDH in the Department of Neurosurgery, Oslo University Hospital, between January and December 2008. The patients were divided into two groups: with unilateral and bilateral CSDH.

The CSDH was diagnosed in all patients by high-resolution CT scanning without and with contrast enhancement on the day of the operation and confirmed by operative findings. All patients underwent the standard surgical procedure in our department under local anaesthesia and propofol sedation. Haematoma evacuation was done by a single burr-hole craniostomy and irrigation with isotonic saline solution, followed by a 1-day period of external continuous closed-system drainage. Patients with bilateral CSDHs received bilateral surgery in one sitting. Postoperatively, they were kept in bed until the drain had been removed. Any anti-aggregant and anticoagulant therapy was temporarily discontinued upon admission (except one patient with mechanical heart valves) and re-established 4 weeks after operation. If the international normalised ratio (INR) was preoperatively more than 1.5 in patients with anticoagulant therapy, the effect of medication was reversed before surgery.

Follow-up CT scans of every patient were performed 24 h after surgery to assess the amount of CSDH evacuation and subsequently at monthly intervals until total disappearance of haematoma cavity and clinically confirmed adequate treatment, but not longer than 7 months. If neurological deficit persisted or clinical deterioration occurred, CT scanning was performed earlier. Follow-up CT scans were repeatedly evaluated for changes of thickness and midline displacement, disappearance of haematoma cavity and neomembranes, and compression signs (e.g. cortical flattening, ventricular and cisternal compression).

If serial CT scanning during the 7 months’ follow-up period revealed increased subdural collection and brain compression on the operated side compared with CT findings at the 1st postoperative day (radiographic recurrence) and neurological symptoms persisted or reappeared (symptomatic recurrence) then a relapse of CSDH was diagnosed and the patient re-operated on [13].

Characteristic CT scan findings of CSDHs considered in this analysis included: (1) site (unilateral or bilateral); (2) intracranial extension (convexity or cranial base type); (3) status of basal cisterns (intact or partly or globally compressed); (4) thickness (preoperative and postoperative); (5) midline displacement (preoperative and postoperative); (6) density appearance; (7) enhancement of the inner membrane and the intrahaematomal trabecules; (8) preoperative volume; (9) residual rinsing fluid volume in haematoma cavity on the 1st postoperative day; (10) residual air volume in haematoma cavity on the 1st postoperative day; (11) residual total haematoma cavity volume on the 1st postoperative day (i.e. the sum of residual rinsing fluid and residual air volumes in haematoma cavity); (12) total disappearance of subdural space and midline shift on postoperative follow-up CT scans.

According to the intracranial extension, the CSDHs were classified into three types [17]: convexity type (localised at the convexity without involvement of the cranial base); cranial base type (extended from convexity to the cranial base); interhemispheric type (extended from convexity to the interhemispheric fissure). The preoperative CT scans were classified into two groups according to the status of the basal cisterns: CSDHs that appeared cerebral cortical and ventricular compression but with intact basal cisterns and those with the basal cisterns partly or globally compressed.

The preoperative and postoperative thickness of CSDH was measured on each CT scan, indicating the maximum in-plane thickness of the haematoma [24, 27]. The midline displacement on pre-and postoperative CT scans was measured as the distance from the septum pellucidum point between the anterior horns of the lateral ventricles to a perpendicular line connecting the anterior and posterior insertions of the falx cerebri. Marked midline displacement was defined as a midline shift measuring more than 5 mm on CT scans. Preoperative contrast CT scans were assessed with regard to the enhancement of the inner membrane and visible intrahaematomal trabecules as not, moderately or markedly enhanced.

**Fig. 1:**
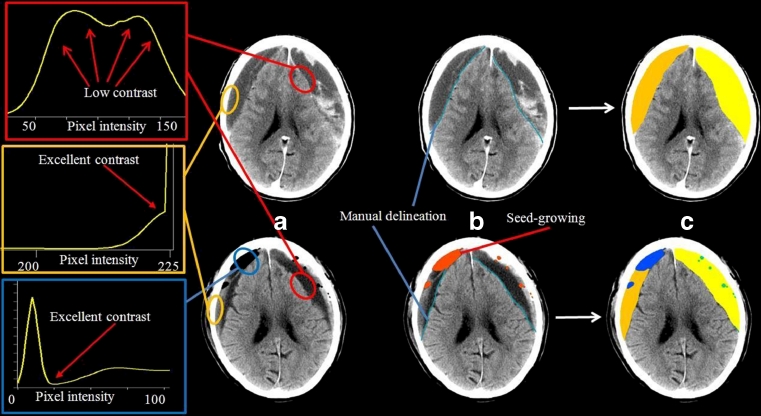
The segmentation and annotation of fluid and air volumes in preoperative and postoperative CT scans. *Upper row*: preoperative images. *Lower row*: postoperative images. Curves to the far left show typical intensity histograms for important image contrasts: brain/haematoma fluid/residual rinsing fluid (*red rectangle*); skull/all other tissues (*orange rectangle*); air/all other tissues (*blue rectangle*). **a** Representative preoperative and postoperative image sets; **b** manual delineation (*blue line*) and semi-automatic seed-growing of air pockets (*red fill*); **c** appearance after region-growing-based fill-in to generate areas with fluid and air, left and right

Estimation of the preoperative volume of CSDH, residual rinsing fluid and air volumes in the haematoma cavity and consequently residual total haematoma cavity volume on the 1st postoperative day was determined from the baseline and postoperative CT scans using the software tool BrainVoyager QX 2.0. Images were electronically transferred in DICOM format to a dedicated workstation for image analysis. Preoperative CSDH and postoperative residual rinsing fluid in the haematoma cavity were delineated against soft tissues using the freehand selection tool on each 2.5– to 5-mm tomographic slice of every CT image series by a single experienced neurosurgeon (M.S.). The area between the demarcation and the skull was filled using a seed-growing algorithm. Delineation of air required no manual delineation due to excellent image contrast, and was instead performed using a manually placed starting and the same seed-growing algorithm as for the CSDH. Voxel dimensions from all individual scans were extracted from the DICOM image header and used to calculate preoperative CSDH metric volumes of left and right, postoperative rinsing fluid and air volumes. The segmentation and estimation procedures are summarised as a cartoon in Fig. 1.

**Fig. 2:**
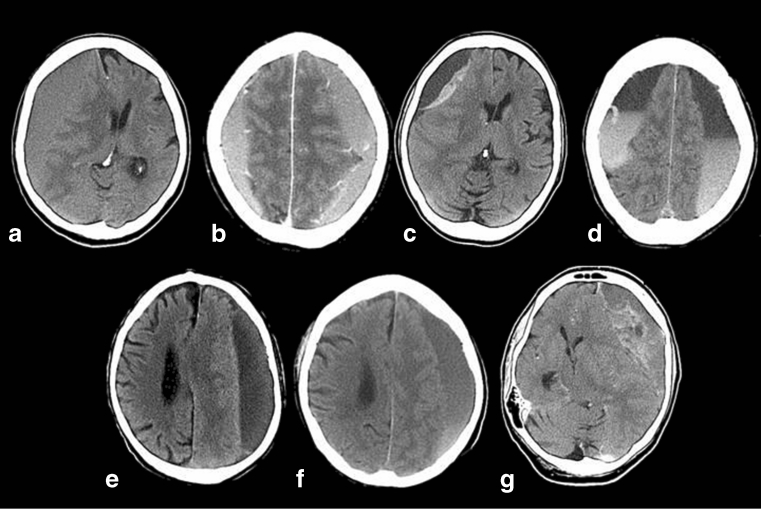
Representative CT densities of CSDHs according to the classification described by Nakaguchi et al. [17]. *Upper row*: densities of haematoma with high recurrence rate: **a** isodense; **b** hyperdense; **c** laminar; **d** separated. *Lower row*: densities of haematoma with low recurrence rate: **e** hypodense; **f** gradation; **g** trabecular

As illustrated in Fig. 2, the imaging appearance of CSDHs based on the density changes was assessed using the classification described by Nakaguchi et al. [17] into four types: the homogeneous (including hypodense, isodense, and hyperdense subtypes), laminar, separated, trabecular and gradation subtype.

### Statistical analyses

Data were described using mean (standard deviation) or number of patients (percentage) for continuous or categorical variables, respectively. Univariate analysis was performed with independent sample *t*-test or chi-squared test as appropriate. Analysis of risk factors for recurrence of CSDH was done using univariate (sometimes denoted as simple regression model) and multivariate analysis (sometimes denoted as multiple regression model). The relation of these risk factors to recurrence was expressed as relative risk rate (RR) with 95 % confidence intervals estimated by Poisson regression. Variables in the final model were selected according to a stepwise method and those deemed to have importance were included. The prognostic ability of preoperative and postoperative volume was assessed using receiver-operating characteristic curves (ROC curves). All statistical analyses were conducted with PASW Statistics 18 (IBM Corporation, Armonk, New York, USA) and considered statistical significant if the *p* value was < 0.05.

## Results

### The patient population and recurrence rates

**Fig. 3:**
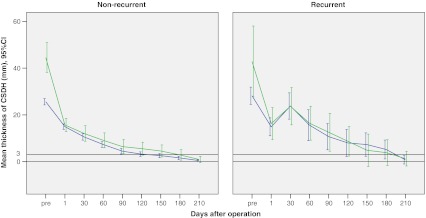
Line plots of the maximum in-plane thickness of CSDH (mean and 95 % CI) assessed by CT scan throughout the study period in non-recurrent and recurrent cases from unilateral (*blue lines*) and bilateral (*green lines*) groups. The thickness of haematoma membrane at 3 mm is indicated with a separate *black horizontal line*

The study consisted of 107 patients with CSDH: 72 males (67.3 %) and 35 females (32.7 %) with mean age 72.1 (± 12.8) years. Eighty-four (78.5 %) patients had unilateral and 23 (21.5 %) bilateral haematomas. In this series, 15.9 % (17 out of 107) patients experienced recurrence of CSDH. Specifically, recurrence was found in 11.9 % (10 out of 84) of patients in the unilateral group and in 30.4 % (7 out of 23) of patients in the bilateral group (Table 1). The difference was statistically significant (*p* = 0.031). Interestingly, as illustrated in Fig. 3, all seven patients with recurrence in the bilateral group had a recurrence on one side only. The mean interval between the initial operation and the re-operation was 30.4 (± 19.8) days. There was no mortality in these 107 patients during the follow-up period.

**Table 1:** Univariate analysis of thickness and midline displacement related to recurrence in 84 patients with unilateral and 23 with bilateral CSDH

CT features	Patients with unilateral CSDH *N* = 84 (100.0 %)			Patients with bilateral CSDH *N* = 23 (100 %)			All patients *N* = 107 (100 %)		
	Recurrence	No recurrence	*p*-value	Recurrence	No recurrence	*p*-value	Recurrence	No recurrence	*p*-value
Number of patients (%)	10 (11.9 %)	74 (88.1 %)		7 (30.4 %)	16 (69.6 %)		17 (15.9 %)	90 (84.1 %)	
Mean in-plane maximum thickness (SD) (mm)									
Preoperative	28.2 (5.1)	25.6 (5.8)	0.191	46.4 (17.6)	44.6 (11.9)	0.767	34.2 (13.1)	29.0 (10.3)	0.068
Postoperative ^a^	14.9 (5.4)	14.9 (5.1)	0.960	18.5 (10.8)	15.7 (5.3)	0.410	15.5 (6.1)	15.1 (5.1)	0.796
Mean maximum midline displacement (SD) (mm)									
Preoperative	12.5 (3.7)	9.4 (4.3)	0.032	2.7 (2.9)	3.1 (2.9)	0.797	8.8 (5.7)	8.2 (4.8)	0.652
Postoperative ^a^	7.3 (2.2)	5.7 (2.3)	0.049	3.5 (2.1)	3.8 (.8)	0.779	4.9 (3.7)	3.9 (3.2)	0.265
Preoperative marked midline displacement									
≤ 5 mm	0 (0 %)	21 (100 %)	0.052	5 (25.0 %)	15 (75.0 %)	0.144	5 (12.2 %)	36 (87.8 %)	0.410
> 5 mm	10 (15.9 %)	53 (84.1 %)		2 (66.7 %)	1 (33.3 %)		12 (18.2 %)	54 (81.8 %)	
Postoperative marked midline displacement ^a^									
≤ 5 mm	3 (5.5 %)	52 (94.5 %)	0.012	7 (30.4 %)	16 (69.6 %)		10 (12.8 %)	68 (87.2 %)	0.155
> 5 mm	7 (24.1 %)	22 (75.9 %)		0			7 (24.1 %)	22 (75.9 %)	

Values are expressed as mean (standard deviation) or number of patients (percentage); *N* number of patients

^a^ On 1st postoperative day

### Associations between preoperative CT characteristics and recurrence rate

The preoperative intracranial extension of CSDH, preoperative status of basal cisterns, enhancement of inner haematoma membrane or intrahaematomal trabecules were not significantly associated with the recurrence on univariate analysis. Likewise, the preoperative maximum in-plane thickness of CSDH in each group and in the entire series was found not to be significantly associated with the recurrence rate (Table 1).

On univariate analysis, the recurrence of CSDH was significantly associated with the preoperative maximum midline displacement in the unilateral group (*p* = 0.032), but not in the bilateral group or throughout the series (Table 1). Similarly, the recurrence was associated with the preoperative marked midline displacement in the unilateral group (*p* = 0.052), but not in the bilateral or in the entire series (Table 1).

Statistical analysis indicated that the recurrence of CSDH was significantly affected by the imaging appearances based on haematoma densities throughout the series (*p* = 0.023) (Table 2). Classification of the CSDHs into two main pre-defined groups by combining the isodense, hyperdense, laminar and separated densities (46 patients), in one group, and the hypodense, gradation and trabecular densities (61 patients) in another group showed that the recurrence rate in the former group 30.4 % (14 out of 46) patients was significantly higher than those in the latter group 4.9 % (3 out of 61) (*p* = 0.003).

**Table 2:** Univariate analysis of CT densities related to recurrence in 84 patients with unilateral and 23 with bilateral CSDH

CT features	Patients with unilateral CSDH *N* = 84 (100.0 %)			Patients with bilateral CSDH *N* = 23 (100 %)			All patients *N* = 107 (100 %)		
	Recurrence	No recurrence	*p* value	Recurrence	No recurrence	*p* value	Recurrence	No recurrence	*p* value
Number of patients (%)	10 (11.9 %)	74 (88.1 %)		7 (30.4 %)	16 (69.6 %)		17 (15.9 %)	90 (84.1 %)	
CT densities									
Hypodense	2 (6.7 %)	28 (93.3 %)	0.203	0 (0 %)	3 (100 %)	0.235	2 (6.1 %)	31 (93.9 %)	0.023
Isodense	4 (26.7 %)	11 (73.3 %)		0 (0 %)	1 (100 %)		4 (25.0 %)	12 (75.0 %)	
Hyperdense	2 (28.6 %)	5 (71.4 %)		3 (50.0 %)	3 (50.0 %)		5 (38.5 %)	8 (61.5 %)	
Laminar	1 (25.0 %)	3 (75.0 %)		2 (50.0 %)	2 (50.0 %)		3 (37.5 %)	5 (62.5 %)	
Gradation	0 (0 %)	6 (100 %)		0 (0 %)	3 (100 %)		0 (0 %)	9 (100 %)	
Separated	0 (0 %)	6 (100 %)		2 (66.7 %)	1 (33.3 %)		2 (22.2 %)	7 (77.8 %)	
Trabecular	1 (6.2 %)	15 (93.8 %)		0 (0 %)	3 (100 %)		1 (5.3 %)	18 (94.7 %)	

Values are expressed as number of patients (percentage); *N* number of patients

Univariate analysis showed that the recurrence was significantly associated with the preoperative haematoma volume in the unilateral group (*p* = 0.007) and in the entire series (*p* = 0.003) (Table 3). The prognostic ability of the preoperative haematoma volume in the entire series was assessed with a ROC curve. The area under the ROC curve was 0.71 (95 % CI 0.58–0.84, *p* = 0.007). A cut-off to give an adequate high sensitivity was set to 115 ml. This cut-off value gave a sensitivity of 88.2 % (95 % CI 65.7–96.7) and specificity of 38.2 % (95 % CI 28.8–48.6). In our sample of patients the probability of no-recurrence was estimated to 94.4 % (95 % CI 81.7–98.5) if preoperative haematoma volume was below 115 ml. This implies, as illustrated in Fig. 5a, that patients with preoperative haematoma volume under 115 ml have a low probability of recurrence.

**Table 3:** Univariate analysis of volumes related to recurrence in 84 patients with unilateral and 23 with bilateral CSDH

CT features	Patients with unilateral CSDH *N* = 84 (100.0 %)			Patients with bilateral CSDH *N* = 23 (100 %)			All patients *N* = 107 (100 %)		
	Recurrence	No recurrence	*p*-value	Recurrence	No recurrence	*p*-value	Recurrence	No recurrence	*p*-value
Number of patients (%)	10 (11.9 %)	74 (88.1 %)		7 (30.4 %)	16 (69.6 %)		17 (15.9 %)	90 (84.1 %)	
Volumes									
Preoperative haematoma volume (ml) ^a^	176.3 (47.7)	131.1 (48.3)	0.007	236.9 (119.2)	213.9 (83.4)	0.599	201.2 (86.9)	146.0 (64.2)	0.003
Postoperative residual fluid volume (ml) ^b^	109.7 (45.6)	66.5 (33.3)	< 0.001	148.7 (93.4)	128.6 (47.1)	0.496	125.7 (69.5)	77.6 (43.0)	0.013
Postoperative residual air volume (ml) ^c^	18.9 (11.1)	19.8 (19.3)	0.839	43.6 (27.5)	31.8 (35.8)	0.448	29.1 (22.6)	21.9 (23.3)	0.245
Postoperative residual cavity volume (ml) ^d^	128.6 (45.9)	86.3 (39.9)	0.003	192.3 (90.3)	160.4 (68.1)	0.360	154.8 (72.7)	99.5 (53.8)	0.007

Values are expressed as number of patients (percentage) or mean (standard deviation); *N* number of patients

^a^ Mean (SD) preoperative volume in unilateral or sum of both sides in bilateral haematoma

^b^ Mean (SD) postoperative residual fluid volume in haematoma cavity in unilateral or both sides sum in bilateral haematoma on 1st postoperative day

^c^ Mean (SD) postoperative residual air volume in haematoma cavity in unilateral or both sides sum in bilateral haematoma on 1st postoperative day

^d^ Mean (SD) postoperative sum of residual fluid and air volumes in haematoma cavity on 1st postoperative day

### Associations between postoperative CT findings and recurrence rate

The thickness of persistent haematoma cavity on the 1st postoperative day in each group and throughout the series was not significantly associated with the recurrence in univariate analysis (Table 1). The preoperative maximum thickness of haematoma and the postoperative changes of thickness of residual haematoma cavity throughout the study period are illustrated in Fig. 3. Thus Fig. 3 shows that:

1. In both non-recurrent and recurrent cases, the thickness of residual haematoma cavity on the 1st postoperative day was significantly reduced in the unilateral and bilateral groups when compared with the preoperative values (*p* < 0.001).
2. In non-recurrent cases, the thickness of residual haematoma cavity gradually decreased postoperatively and the residual CSDH cavity totally disappeared in the unilateral group by the 120 days and in the bilateral by the 180, but the changes after first postoperative day were not significant.
3. In recurrent cases, the thickness of residual haematoma cavity at the time of diagnosis of recurrence was significantly increased compared to thickness on 1st postoperative day in both the unilateral and bilateral groups (*p* < 0.001). After re-operation, the thickness of residual haematoma cavity gradually decreased and the residual haematoma cavity totally disappeared in all cases of the unilateral and bilateral groups by 190 postoperative days.

Three months after initial operation, the total disappearance of the haematoma cavity was confirmed in 45.6 % (41 out of 90) non-recurrent patients and 17.6 % (3 out of 17) recurrent. The difference was statistically significant (*p* = 0.037).

Univariate analysis showed significant association between the recurrence of haematoma and the maximum midline displacement on the 1st postoperative day in the unilateral group (*p* = 0.049), but not in the bilateral group or in the entire series (Table 1). Similarly, the recurrence was significantly associated with the marked midline displacement on the 1st postoperative day in the unilateral group (*p* = 0.012), but not in the bilateral group or throughout the series (Table 1).

**Fig. 4:**
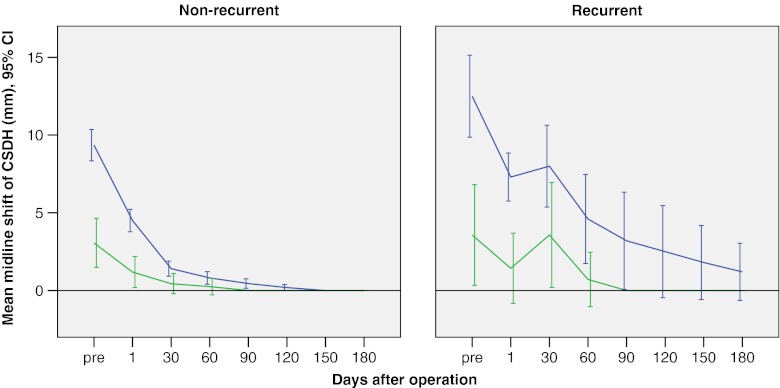
Line plots of the maximum midline displacement (mean and 95 % CI) assessed by CT scan throughout the study period in non-recurrent and recurrent cases from unilateral (*blue lines*) and bilateral (*green lines*) groups

The preoperative maximum midline displacement and the postoperative changes of displacement throughout the study period are illustrated in Fig. 4. Thus Fig. 4 shows that:

1. In both non-recurrent and recurrent cases, the postoperative midline displacement on the 1st postoperative day was significantly reduced in both the unilateral and bilateral groups when compared with the preoperative values (*p* < 0.001).
2. In non-recurrent cases, the midline displacement gradually decreased postoperatively and totally disappeared in all cases in the unilateral group by 120 days and in the bilateral by 70 days.
3. In recurrent cases, the postoperative midline displacement at the time of diagnosis of recurrence was not significantly increased when compared with the midline shift of the 1st postoperative day in both the unilateral and the bilateral groups (*p* = 0.550 and *p* = 0.228 respectively). After re-operation, the midline displacement gradually decreased and totally disappeared in all cases of the bilateral group by 90 postoperative days, but this required more than 6 months in the unilateral group.

Three months after initial operation, the total disappearance of midline displacement was confirmed in 91.1 % (82 out of 90) non-recurrent patients and in 76.5 % (13 out of 17) recurrent patients. The difference was statistically significant (*p* = 0.040).

The residual total haematoma cavity volume on the 1st postoperative day in both the unilateral and bilateral groups and in the entire series was significantly decreased compared with the preoperative haematoma volume (*p* < 0.001). The postoperative recurrence was statistically significantly associated with the residual rinsing fluid volume in the haematoma cavity and the residual total haematoma cavity volume on the 1st postoperative day in the unilateral group (*p* < 0.001 and *p* = 0.003 respectively) and in the entire series (*p* = 0.013 and *p* = 0.007 respectively) (Table 3). However, the recurrence was not found associated with the residual air volume in the haematoma cavity on the 1st postoperative day (Table 3).

**Fig. 5:**
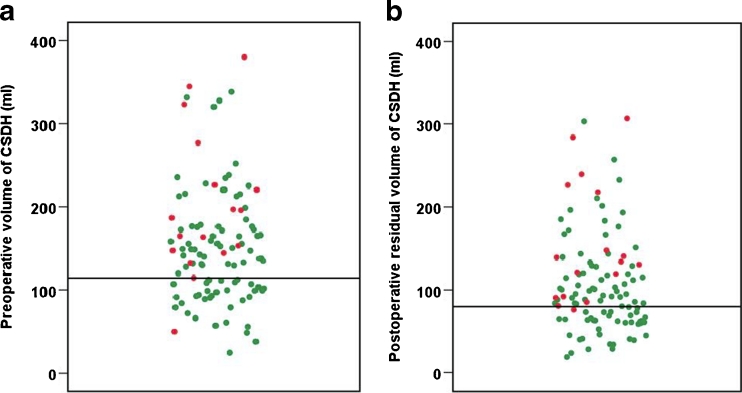
Prognosis of postoperative no-recurrence of chronic subdural haematoma with respect to the distribution of preoperative and postoperative volumes. *Green points* non-recurrent cases; *red points* recurrent cases. Separate *black horizontal lines* indicate cut-off values. **a** The probability of no recurrence was estimated to 94.4 % (95 % CI 81.7–98.5) if the preoperative haematoma volume was under 115 ml. **b** The probability of no-recurrence was estimated to be 97.4 % (95 % CI 86.5–99.5) if the residual total haematoma cavity volume on the 1st postoperative day was under 80 ml

The prognostic ability of the residual total haematoma cavity volume on the 1st postoperative day on recurrence throughout the series was assessed with a ROC curve. The area under the ROC curve was 0.76 (95 % CI 0.64–0.87, *p* = 0.001). A cut-off to give an adequate high sensitivity was set to 80 ml. This cut-off value gave a sensitivity of 94.1 % (95 % CI 73.0–98.9) and specificity of 41.1 % (95 % CI 31.5–51.4). The negative predictive value was estimated in this cohort to be 97.4 % (95 % CI 86.5–99.5). In other words, as illustrated in Fig. 5b, if the patient at the 1st postoperative day had a residual total haematoma cavity volume less than 80 ml, the probability of no recurrence was 97.4 % in our cohort.

### The results of the multivariate analysis

After eliminating highly correlated variables to avoid colinearity effects in the statistical model, the following items were selected based on a stepwise statistical procedure and adopted as predictors on postoperative recurrence: (1) site of haematoma (bilateral), (2) preoperative haematoma volume (ml), (3) preoperative maximum thickness of haematoma (mm), (4) CT densities of haematoma indicating greater tendency to re-bleeding and high protein exudation rates (isodense, hyperdense, laminar, separated), (5) preoperative maximum midline displacement (mm), and (6) residual total haematoma cavity volume on the 1st postoperative day (ml). The relative risk (RR) for these variables shown in Table 4 is the effect of 1 unit increase.

**Table 4:** Results of univariate (simple regression model) and a multivariate (multiple regression model) analyses of radiological variables adopted as predictors on postoperative recurrence of CSDH

Predictor variables		Univariate model *N* = 107 (100.0 %)			Multivariate model *N* = 107 (100 %)		
	Unit	RR	95 % CI	*p* value	RR	95 % CI	*p* value
Site of CSDH	if bilateral	2.526	0.962–6.636	0.060			
Preoperative haematoma volume ^a^	ml	1.007	1.002–1.013	0.008	1.005	1.000–1.011	0.052
Preoperative haematoma thickness ^b^	mm	1.030	0.994–1.068	0.105			
CT densities ^c^	if isodense, hyperdense, laminar or separated	6.087	1.749–21.181	0.005	5.106	1.438–18.127	0.012
Preoperative midline displacement	mm	1.018	0.923–1.122	0.722			
Postoperative residual haematoma cavity volume ^d^	ml	1.009	1.003–1.015	0.002	1.007	1.001–1.013	0.022

*N* number of patients (percentage), *RR* relative risk, *CI* confidence interval

^a^ Preoperative volume in a unilateral haematoma or the sum of both sides in a bilateral haematoma

^b^ Preoperative maximum thickness in a unilateral haematoma or the sum of maximum thickness in a bilateral haematoma

^c^ Densities indicating greater tendency to re-bleeding and high protein exudation rates

^d^ Sum of residual fluid and air volumes in the haematoma cavity on the 1st postoperative day after removal of the drainage

We performed a multivariate analysis with a Poisson regression model (multiple regression analysis) and found that the preoperative haematoma volume, the isodense, hyperdense, laminar and separated CT densities and the residual total haematoma cavity volume on the 1st postoperative day were independent predictors for the postoperative recurrence of CSDH (Table 4). Other selected radiological variables were found not to have a significant prediction on the recurrence (Table 4). It is warranted to validate prognostic ability of the multiple Poisson regression model (multivariate analysis) using other patient data. Three predictors in a model with only 17 events (recurrences) may cause some risk of statistical over-fitting.

## Discussion

CT scanning of a patient with CSDH provides a wealth of information on the intracranial status and remains the most important radiological diagnostic investigation for this disorder. The CT scan characteristics have been extensively studied as prognostic factors for recurrence of CSDH; however, findings were commonly inconsistent. We therefore assessed the relationship of the CT scan features and recurrence of CSDH in our series by performing univariate (i.e. simple Poisson regression model) and multivariate (i.e. multiple Poisson regression model) analyses.

Amongst several CT features of CSDH evaluated in this study, we found that three were predictors for the postoperative recurrence: (1) the preoperative haematoma volume; (2) the isodense, hyperdense, laminar and separated CT densities; (3) the residual total haematoma cavity volume on the 1st postoperative day after removal of the drainage.

The size of a CSDH at the time of radiological diagnosis can be impressive. Estimation of the preoperative haematoma size by measuring of the volume by XYZ/2 [28, 29] and computer-assisted volumetric techniques [28] has been reported; however, this CT characteristic has not been investigated sufficiently in patients with CSDH. To our knowledge, this study is the first to prospectively investigate the preoperative haematoma volume and the volume of residual total haematoma cavity on the 1st postoperative day as risk factors for recurrence.

The preoperative haematoma volume determined using a computer-assisted volumetric technique was a powerful estimator of haematoma size. We found that the preoperative haematoma volume was a predictor of postoperative recurrence. Additionally, we found that the prognosis of no-recurrence was very high (94.4 %) in our cohort if the preoperative haematoma volume was under 115 ml, consistent with a general consideration that patients with large CSDH are at increased risk for the recurrence [10, 16, 24, 29]. Increased size of haematoma is attributed to brain atrophy associated with ageing, which may provide the CSDH with a potential space in which to grow [30]. Furthermore, it has been speculated that brain atrophy may lead to inappropriate brain re-expansion postoperatively and thereby create the potential for re-accumulation of the haematoma [4, 16].

Postoperatively, after the burr-hole irrigation procedure, the haematoma cavity is filled by rinsing fluid and introduced air. The computer-assisted volumetric technique used in this study was a potent estimator of residual total haematoma cavity volume on the 1st postoperative day. We found that the residual total haematoma cavity volume on the 1st postoperative day was a predictor of postoperative recurrence. This finding corroborating a general consideration that persistence of the haematoma space postoperatively may predispose recurrence because it may inhibit brain re-expansion and prevent reduction of the haematoma cavity [4, 27, 31]. Furthermore, we found that the prognosis of no-recurrence was very high (97.4 %) in our cohort if the residual total haematoma cavity volume at the 1st postoperative day after removal of drainage was under 80 ml. This finding suggests that the drainage period of 24 h postoperatively used in this series may not be sufficient and it has been reported that prolonged duration of drainage for at least 3 full days may reduce the postoperative recurrence [9].

The postoperative residual air accumulation in the haematoma cavity has been reported to be a risk factor for recurrence [4, 19, 27]. Therefore, complete replacement of CSDH fluid by normal saline to prevent influx of air into the haematoma cavity has been suggested [4, 19]. Additionally, it is also recommended to place the tip of drainage catheter in the frontal convexity of the haematoma cavity to remove subdural air during and/or after surgery [27, 32]. However, in this series, the recurrence was not affected by the amount of residual air in the haematoma cavity on the 1st postoperative day.

The imaging appearance of the CSDH on CT scans may help identify fresh blood from re-bleeding, suggest the age of the haematoma, and reflect upon the protein concentration from plasma exudation [17, 20, 33, 34]. Thus, isodense, hyperdense, laminar and separated appearances are considered to have a greater tendency to re-bleeding [16–19] and high protein exudation rates [20]. Our data indicated that these appearances on CT scans were predictors of the postoperative recurrence. These findings correspond well to some results reported in literature. Thus, a high recurrence rate was reported in the hyperdense subtype [2, 15, 19], laminar type [17] and the separated type [13, 17, 19], whereas the recurrence of the trabecular type [8, 13, 17] and hypodensity subtype was low [2, 13, 19]. However, Mori et al. [4] and Tsai et al. [7] could not find a positive correlation between imaging appearance based on CT densities and recurrence.

## Conclusions

The preoperative volume of CSDH, the isodense, hyperdense, laminar and separated CT densities and the residual total haematoma cavity volume on the 1st postoperative day after removal of the drainage were radiological predictors of postoperative recurrence. These findings from CT imaging may help to identify patients at risk for postoperative recurrence. The CT characteristics described may be useful for tailoring of individual treatment strategies for patients with this disorder in future clinical trials.
